# Semi-annual seasonal pattern of serum thyrotropin in adults

**DOI:** 10.1038/s41598-019-47349-4

**Published:** 2019-07-25

**Authors:** Daniele Santi, Giorgia Spaggiari, Giulia Brigante, Monica Setti, Simonetta Tagliavini, Tommaso Trenti, Manuela Simoni

**Affiliations:** 1 Unit of Endocrinology, Department of Biomedical, Metabolic and Neural Sciences, University of Modena and Reggio Emilia, Modena, Italy; 2 Unit of Endocrinology, Department of Medical Specialties, Azienda Ospedaliero-Universitaria of Modena, OCSAE, Modena, Italy; 3 Service of Clinical Engineering, Azienda Ospedaliero-Universitaria of Modena, Modena, Italy; 4 Department of Laboratory Medicine and Anatomy Pathology, Azienda USL of Modena, Modena, Italy

**Keywords:** Multihormonal system disorders, Thyroid diseases

## Abstract

Circannual rhythmicity in thyroid-stimulating hormone (TSH) secretion is proposed, whereas evidences on seasonal peripheral thyroid hormones’ fluctuation are contradictory. This study was designed to evaluate hypothalamic-pituitary-thyroid (HPT) seasonal secretion pattern using a big data approach. An observational, retrospective, big data trial was carried out, including all TSH measurements performed in a single laboratory between January 2010 and December 2017. A large dataset was created matching TSH data with patients’ age, gender, environmental temperature exposure, and free triiodothyronine (fT3) and free thyroxine (fT4) when available. The trend and seasonal distributions were analysed using autoregressive integrated moving average models. A total of 1,506,495 data were included in the final database with patients mean age of 59.00 ± 18.44 years. The mean TSH serum levels were 2.08 ± 1.57 microIU/mL, showing a seasonal distribution with higher levels in summer and winter seasons, independently from age, gender and environmental temperatures. Neither fT3 nor fT4 showed a seasonal trend. TSH seasonal changes occurred independently from peripheral thyroid hormone variations, gender, age and environmental temperatures. Although seasonal TSH fluctuation could represent a residual ancestral mechanism to maintain HPT homeostasis, the underlying physiological mechanism remains unclear and specific studies are needed to clarify its impacting role in humans.

## Introduction

Hormone secretion generally follows circadian or seasonal rhythms. In humans, a multi-frequency time structure is detected in the hypothalamic-pituitary (HP)-gonadal, HP-thyroid (HPT) and HP-adrenal axes. Considering the HPT axis, a circadian rhythm is observed at each level, from hypothalamic neurons to the pituitary gland and, finally, the thyroid^1,2^.

In addition to the circadian rhythm, some authors suggested also a seasonal variation of HPT functionality. Recent studies have turned on the lights on this topic^3,4^ that still remains controversial since contrasting data emerged^5^. Several studies have been performed since the beginning of the 80 s of the last century, showing a circannual thyroid-stimulating hormone (TSH) variation, with highest levels in the winter season^6–10^. A clear demonstration of seasonal variations in peripheral thyroid hormones was reached so far, suggesting that circannual variations of TSH should be independent of peripheral hormone fluctuation^1,10^. However, several authors detected higher free triiodothyronine (fT3) levels during winter, in correlation with ambient temperature and day-light reduction^11–13^. On the contrary, a recent study depicted a significant decrease in peripheral thyroid hormones when the highest TSH levels were detected, suggesting a strict interconnection of all actors involved in the HPT axis^14^.

It is well known that several species, including mammals, manifest repeated seasonal cycles in the presence of fixed light-dark cycles^15–18^. In addition to the photoperiodic circadian clock, another timer was observed and it is commonly called “circannual”, although the periodicity do not always exactly coincide with one year^17^. This hypothetical circannual or seasonal hormone rhythm shows species-specific features, which seem to be synchronized to the seasons^19^. Considering the HPT axis, light stimulus seems to induce TSH secretion and local T3 activation at the pituitary level in animal models, while low temperatures increase circulating levels of T3 to regulate adaptive thermogenesis^20^.

Thus, seasonal variations of HPT functionality are possible but not fully elucidated yet, making it even more difficult to understand why they occur. Several hypothesis have been generated, suggesting a possible influencing role of environmental temperatures^21^, vitamin D deficiency^22^ and variations in iodine intake^11,23–25^.

Considering the contradictory results obtained on this topic so far, this study was designed to evaluate TSH seasonal secretion pattern using a big data approach. The hypothetical TSH fluctuation was studied considering the relationship with peripheral thyroid hormones and the environmental temperature exposure.

## Materials and Methods

An observational, retrospective, big data trial was carried out in the “Ospedale Civile Sant’Agostino Estense” (OCSAE) of Modena, Italy. All methods were carried out in accordance with relevant guidelines and regulations, after the acceptance of the Hospital management.

All the tests performed at OCSAE in the central laboratory of the Department of Clinical Pathology, which serves the entire province with over 700,000 inhabitants, were included in a large database, enclosing 990,904,591 records. This data consisted of biochemical and hormonal parameters. All the TSH serum measurements available in this big database were performed between January 2010 and December 2017. A single large dataset was created uploading TSH measurements, patients’ age, gender, residential address, blood test date and the clinical reason leading to TSH measurement. Moreover, fT3 and free thyroxine (fT4) measurements were added for each patient when available. From clinical practice, since February 2011, fT3 and fT4 were measured only when TSH was above or under normal ranges or when specific clinical requests were provided.

The dataset was then cleaned considering the following inclusion criteria: (i) age between 18 and 100 years, (ii) TSH values between 0.05 and 10.00 microIU/mL with peripheral thyroid hormones within the normal reference ranges, and (iii) no concomitant levothyroxine or thyrostatic treatment as much as known. Moreover, all TSH data associated to fT3 and/or fT4 lower or higher than normal ranges were excluded from dataset. The TSH range used for inclusion was larger than normal range of our laboratory (0.35–4.94 microIU/mL). This choice was the consequence of the well-known limit of the TSH normal range to detect clinical thyroid dysfunction. Indeed, although the analytical performances of TSH immunoassays have been progressively improved, there are still some systematic differences among commercially available methods^26–28^. In particular, multiple factors are known to influence the upper TSH limit and, although several trials tried to detect the most reliable normal TSH ranges, specific cut-offs have not been defined yet^26^. Clinical guidelines suggest to propose levothyroxine therapy in asymptomatic patients only when TSH exceeds 10.00 microIU/mL^29^, since a recent meta-analysis demonstrated an absence of increased mortality and morbidity risk below this threshold^30^. Clinical reason for TSH measurement was used to exclude analyses performed to control the adequacy of therapies impacting thyroid function. Finally, each TSH value was considered *per se* and the final dataset contained also patients evaluated more than one time.

### Thyroid function evaluation

TSH serum levels were measured by chemiluminescent microparticle immunoassay (Abbott Diagnostics, USA), with intra-assay coefficient of variation (CV) of 3.10% and an inter-assay CV of 3.50%. Reference ranges were 0.35–4.94 microIU/mL.

FT3 serum levels were measured by chemiluminescent microparticle immunoassay (Abbott Diagnostics, USA), with intra-assay CV of 2.80% and an inter-assay CV of 3.65%. Reference ranges were 1.70–3.70 pg/mL.

FT4 serum levels were measured by chemiluminescent microparticle immunoassay (Abbott Diagnostics, USA), with intra-assay CV of 3.80% and an inter-assay CV of 5.70%. Reference ranges were 7.00–15.00 pg/mL.

### Environmental exposure

Environmental temperatures were included in the large dataset, considering the place where each patient lived for geo-localization. Environmental exposure was evaluated considering both the maximum and the minimum temperatures experienced by patient in the day the blood sample was taken.

Temperature data were obtained using a meteorological model, CALMET, developed at the Hydro Meteorological Service of the Emilia-Romagna environmental protection agency (ARPA) (https://www.arpae.it). The temperature recording unit was geo-localized considering the nearest available site of registration.

### Statistical analysis

Statistical analyses were performed using RStudio Server Open Source Edit Version 0.99.902 2016 and R programming software. The variables distribution was evaluated by Kolmogorov-Smirnov test. The relationship between age and TSH serum levels was evaluated performing Spearman correlation test. Continuous variables distribution differences between groups were evaluated by Mann Whitney U test.

The TSH seasonal fluctuation was evaluated plotting data collected according to the time series distribution. The trend and seasonal distributions were evaluated and the auto-correlations were analysed using the autoregressive integrated moving average (ARIMA) model. ARIMA is a generalization of an autoregressive moving average (ARMA) model, created to better understand the series data distribution. ARIMA models are generally denoted ARIMA (p, d, q), where parameters p, d, and q are non-negative integers, p is the order (number of time lags) of the autoregressive model, d is the degree of differencing (the number of times the data have had past values subtracted), and q is the order of the moving-average model. The auto-ARIMA function was used to select the best model to be applied to describe time series distribution. Finally, the Ljung–Box test was used to detect seasonality, considering whether any of a group of autocorrelations of a time series are different from zero. The seasonal TSH fluctuation was evaluated also considering only TSH serum levels within the normal range of our laboratory. Moreover, TSH fluctuations were evaluated dividing patients according to gender. Seasonal fluctuations were evaluated also for fT4 and fT3 serum levels.

The correlation among TSH, fT4 and fT3 and environmental temperatures was evaluated. Univariate and bivariate statistical analyses were performed and the correlations among continuous variables were evaluated using the Spearman- and the Pearson coefficient, respectively. The quantum geographic information system (QGIS) software was used to evaluate the geographical distribution of all variables.

### Compliance with Ethical Standards

No potential conflicts of interest were present. No humans or animals were directly involved in the study. All procedures performed were in accordance with the ethical standards of the 1964 Helsinki declaration. Considering the retrospective study design and the large number of data extracted, the local Ethics Committee (‘Comitato Etico dell’Area Vasta Emilia Nord’) did not required informed consent from participants included in the study since all data were handled in anonymized form after the approval of the Hospital management (‘Board Aziendale Ricerca e Innovazione’, Azienda Ospedaliero-Universitaria of Modena), which provided access to data.

## Results

Initially, 1,561,614 TSH measurements were included in the dataset, considering 1,022,698 (65.48%) women and 538,916 (34.51%) men. Then, 660,562 fT4 and 660,722 fT3 detections were added. Thus, only in the 42% of the studied cohort, both TSH and fT3 and fT4 levels were available. Thus, the final dataset included 1,237,321 records. Information about maximum and minimum environmental temperatures, registered the day of blood sampling in the station near to the residential address of each subject, were collected.

Considering the overall dataset, the mean age was 58.03 ± 19.51 years (median 59, min 1, max 105). The mean TSH serum levels were 2.35 ± 10.54 microIU/mL (median 1.74, min <0.001, max 694). Mean fT3 serum levels were 2.73 ± 1.35 pg/mL and mean fT4 10.47 ± 7.69 ng/mL.

Among TSH measurements, 29,543 were excluded since the patients’ age was under 18 years. Similarly, 101 measurements were excluded since the patients’ age was above 100 years. Considering TSH serum levels, data comprised between 0.05 and 10.00 microIU/mL were considered. Thus, 22,498 and 3,087 measurements were excluded because, respectively, out of this “arbitrary” range. A total of 1,506,495 data were included in the final database. In the second analysis, TSH data within laboratory normal range were included, for a total of 1,373,575 data.

Consequently, the mean TSH serum level was 2.08 ± 1.57 microIU/mL (median 1.73, min 0.05, max 9.99). In the second analysis, the mean TSH serum level was 1.91 ± 1.47 microIU/mL (median 1.72, min 0.35, max 4.94 microIU/mL). The mean age was 59.00 ± 18.44 years (median 60, min 18, max 99). Both TSH (D = 0.649, p < 0.001) (Supplementary Fig. 1) and age (D = 1, p < 0.001) were not normally distributed.

TSH serum levels were significantly and inversely correlated to patients’ age (Spearman’s Rho −0.067, p < 0.001). TSH serum levels were significantly (p < 0.001) higher in females compared to males (2.18 ± 1.63 versus 1.90 ± 1.44 microIU/mL, respectively). Moreover, women were significantly (p < 0.001) younger than men (58.06 ± 18.80 versus 60.78 ± 17.61 years).

### TSH seasonal change

**Figure 1:**
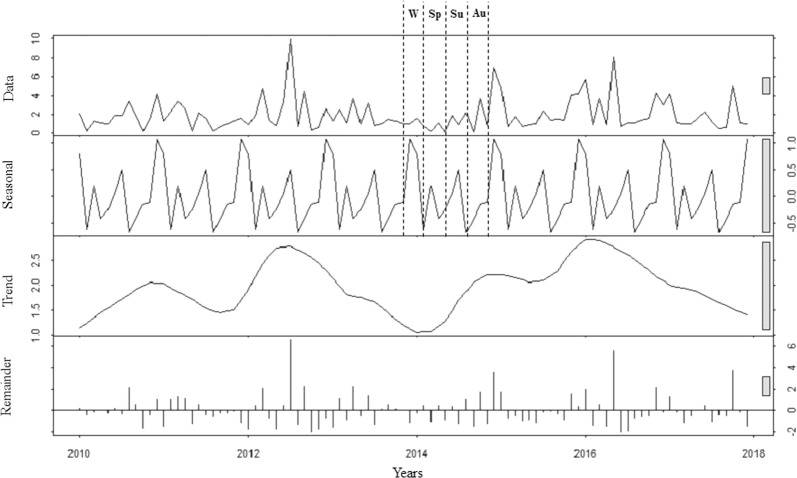
Thyroid-stimulating hormone distribution analysis using ARIMA model (0, 0, 0). The first row shows data distribution; the second row highlights the seasonal pattern of data distribution; the third row shows the data trend across years and the last row the residuals distribution. Au: autumn; Sp: spring; Su: summer; W: winter.

The first row of Fig. 1 shows TSH serum levels distribution along the years. TSH levels were not stationary across years (Dickey-Fuller Test, p < 0.001).

**Figure 2:**
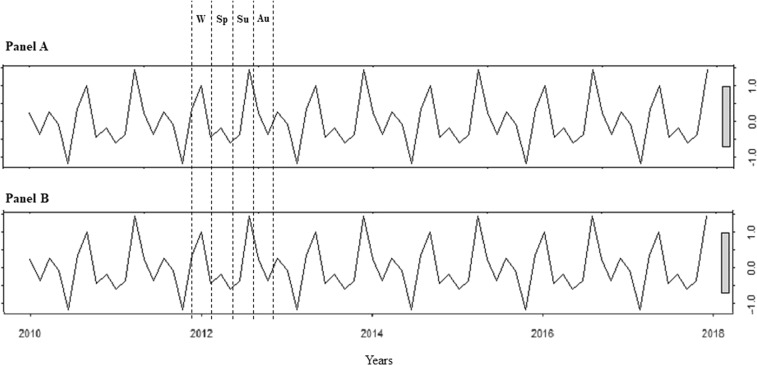
Thyroid-stimulating hormone distribution analysis using ARIMA model (0, 0, 0) in males (panel A) and females (panel B). Au: autumn; Sp: spring; Su: summer; W: winter.

The auto-ARIMA test selected the ARIMA (0, 0, 0) as the best applicable model, depicting following coefficients: sigma^2^ estimated 2.95 with log likelihood = −187.65, Akaike’s information criterion (AIC) = 379.29 and Bayesian information criterion (BIC) = 384.42. From the distribution analysis, TSH did not show a significant trend across years (third row), but rather a seasonal distribution (second row) (Fig. 1). Residuals were equally distributed (fourth row) (Fig. 1). The Box-Ljung test confirmed the significant seasonal distribution of TSH (X-squared = 38.147, degrees of freedom = 8, p-value = 0.042), with higher levels in summer and winter seasons (Fig. 1). Similar seasonality was detected considering males and females separately (p = 0.038 and p = 0.043, respectively) (Fig. 2).

Considering the normal laboratory ranges for TSH, the original dataset was reduced to 91% (1,373,575 data). The seasonal TSH distribution remained when only TSH within the normal range were considered at ARIMA (AIC = 391.91, BIC = 402.17) (Supplementary Fig. 2) and Box-Ljung test (p-value = 0.039).

### Peripheral thyroid hormones seasonal change

**Figure 3:**
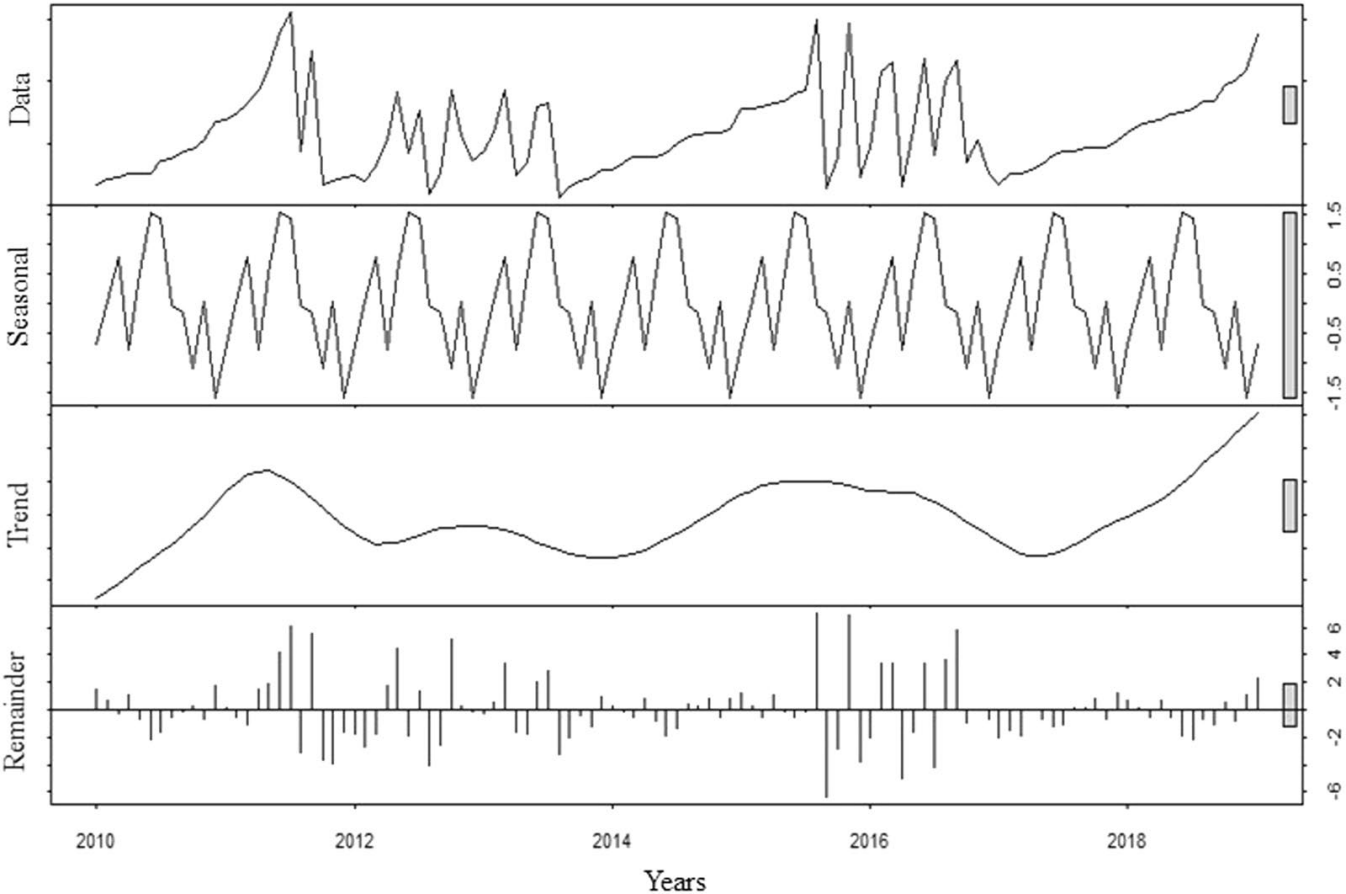
Free thyroxine distribution analysis using ARIMA model (0, 0, 0). The first row shows data distribution; the second row highlights the seasonal pattern of data distribution; the third row shows the data trend across years and the last row the residuals distribution.

Both fT3 and fT4 were not stationary across the years of observation (Dickey-Fuller Test, p < 0.001). Similar to TSH, the auto-ARIMA test selected the ARIMA (0,0,0) as the best applicable model, providing AIC = 698.32 and BIC = 836.33. From the distribution analysis, both fT3 and fT4 showed neither a significant trend across years, nor a seasonal trend (Fig. 3). The Box-Ljung test confirmed the lack of seasonal distribution of both fT4 (X-squared = 9.103, degrees of freedom = 8, p-value = 0.698) and fT3 (X-squared = 3.587, degrees of freedom = 8, p-value = 0.531).

### Environmental temperatures impact

The mean environmental temperatures registered during the years of observation was 14.05 ± 7.83 °C, ranging from −14.5 °C to 39.5 °C. Both maximum and minimum temperatures did not correlate with TSH (Rho = 0.023, p = 0.650 and Rho = 0.026, p = 0.634, respectively), fT3 (Rho = 0.067, p = 0.332 and Rho = 0.070, p = 0.245, respectively), and fT4 (Rho = 0.002, p = 0.933 and Rho = 0.003, p = 0.879, respectively) at linear regression analyses.

## Discussion

For the first time, a big data approach demonstrates seasonal variations of TSH serum levels, with a semi-annual peak, independent from peripheral thyroid hormones, gender, age and environmental temperatures.

The semi-annual TSH serum levels fluctuation shows two specific peaks occurring in summer and winter. A circannual TSH secretion pattern was already suggested both in healthy subjects^1,7^ and in hypothyroid patients receiving constant levothyroxine treatment^6^, although several doubts remain about any possible, peripheral thyroid hormones’ fluctuation. Here, we confirm this seasonal TSH pattern, independent from changes in fT3 and fT4 serum levels, using a big data approach. In the literature, six studies detected seasonal variations in TSH and peripheral thyroid hormones, with highest T3 and T4 levels from September to February^21,31–35^. Considering that our study analysed more than 650,000 fT3 and ft4 measurements, we can support the independency of seasonal variations of TSH from peripheral thyroid hormones. This seasonal secretion pattern is further independent from age and gender.

Serum TSH secretion seems to be seasonally regulated, partially escaping thyroid hormones negative feedback. Accordingly, animal models showed a seasonal different expression of thyroid hormone receptors in brain^36^. Thus, the hypothalamic influence on TSH could be predominant in determining its seasonal pattern. As a confirmation, circadian activation of transcription and translation of genes located in the suprachiasmatic nucleus (SCN) was demonstrated^37^. Moreover, SCN lesions lead to TSH circadian rhythmicity disruption in animal models^38^. Finally, the role of seasonal hormone secretion pattern in neurogenesis and cerebral plasticity has been proposed in animal models^39^.

Animals experience seasonal changes in photoperiod, temperature, and precipitation, adapting their physiology and behaviours, such as growth, migration, hibernation, moulting, and reproduction^40^. These changes are regulated by endocrine rhythms, adapted to photoperiod, environment and seasonal changes^40^. This is more evident for seasonal breeders, but could be relevant also for non-seasonal breeders, such as humans, mice and rats. Here, we confirm that TSH is involved in this seasonal adaptation also in humans, although a relevant influence of environmental temperatures on this “bio-clock” is not evident, differently from what previously suggested^21^. We applied a specific geo-localization process to connect TSH to temperatures, already used in an andrological setting, depicting a significant correlation between semen quality and environment^41,42^. We demonstrated that TSH seasonality is not related to maximum or minimum environmental temperatures. Thus, seasonal TSH rhythm seems to be independent from the environment in which the subject lives. We know that hormonal circannual secretion is needed for several animals. In birds, for example, the photoperiodic adaptation of TSH secretion allows to decrease their body weight, including also a reduction in gonadal volume, and consequently gonadal weight, to maximize flight performance during non-breeding seasons^43^. After that, when long-day favourable conditions are detected, birds re-develop gonads within a few weeks However, in humans, the meaning of TSH seasonality is not yet understood. In particular, thyroid hormone regulation of reproductive function detected in animals^44^ still needs to be confirmed in humans. Thus, future development of seasonality studies, connecting thyroid function and reproduction, could be useful.

Compared to previous studies, our trial has three major strengths. First, we used a big data approach and more than 1.5 million of TSH measurements were evaluated. Indeed, previous trials considered a limited number of subjects, from 26 to a maximum of 8,000^7^. Second, the analysis was performed considering both laboratory normal TSH ranges, as well as a wider clinically-accepted interval, allowing an accurate evaluation of hormone fluctuation. Third, a complex ARIMA model was applied to decompose data distribution across years, evaluating both trend and seasonal changes. Previous trials, indeed, show several pitfalls in data distribution analyses, since the seasonal variation is generally evaluated as difference in mean hormone levels between selected periods of the year^33^, or the result of cosinor functions^1,7,10,21,33^. However, the main limit of our study remains the lack of detailed clinical information about included subjects. Although the information on the reason for TSH measurement allowed us to exclude those taking drugs affecting thyroid function, we could not exclude with certainty all the patients with a history of thyroid dysfunction. A second limit is that not every TSH measurement is connected to fT3 and fT4 levels. Even if this is widely accepted in clinical practice^35,45^, it leads to incomplete information about HPT functionality.

In conclusion, we applied big data approach to the evaluation of HPT axis seasonality, depicting a semi-annual secretion pattern of TSH, independent from fT3 and fT4 changes, age, gender and environmental temperatures. Thus, seasonal TSH rhythm seems to be predominantly regulated at the hypothalamic level, maybe as a residual ancestral mechanism to maintain HPT homeostasis. Although this seasonality is clearly demonstrated, its role remains unclear and specific studies, possibly connecting thyroid and reproduction, could be useful to better understand the underlying physiological mechanisms.

## Supplementary information

Supplementary figures 1 and 2

## Data Availability

The datasets generated during and analysed during the current study are available from the corresponding author on reasonable request.

## References

[1] Maes M (1997). Components of biological variation, including seasonality, in blood concentrations of TSH, TT3, FT4, PRL, cortisol and testosterone in healthy volunteers. Clinical endocrinology.

[2] Angeli A, Gatti G, Sartori ML, Masera RG (1992). Chronobiological aspects of the neuroendocrine-immune network. Regulation of human natural killer (NK) cell activity as a model. Chronobiologia.

[3] Das G (2018). Seasonal variation of vitamin d and serum thyrotropin levels and its relationship in a euthyroid caucasian population. Endocrine practice: official journal of the American College of Endocrinology and the American Association of Clinical Endocrinologists.

[4] Gullo D (2017). Seasonal variations in TSH serum levels in athyreotic patients under L-thyroxine replacement monotherapy. Clinical endocrinology.

[5] Ehrenkranz J (2015). Circadian and Circannual Rhythms in Thyroid Hormones: Determining the TSH and Free T4 Reference Intervals Based Upon Time of Day, Age, and Sex. Thyroid: official journal of the American Thyroid Association.

[6] Konno N, Morikawa K (1982). Seasonal variation of serum thyrotropin concentration and thyrotropin response to thyrotropin-releasing hormone in patients with primary hypothyroidism on constant replacement dosage of thyroxine. The Journal of clinical endocrinology and metabolism.

[7] Simoni M (1990). Circannual rhythm of plasma thyrotropin in middle-aged and old euthyroid subjects. Hormone research.

[8] Guagnano MT, Angelucci E, Del Ponte A, Boni R, Sensi S (1984). Circadian and circumannual variations in the level of plasma TSH and prolactin in healthy adult males. Bollettino della Societa italiana di biologia sperimentale.

[9] Tarquini B, Halberg F, Seal US, Benvenuti M, Cagnoni M (1981). Circadian aspects of serum prolactin and TSH lowering by bromocriptine in patients with prostatic hypertrophy. The Prostate.

[10] Bellastella A (1984). Circannual rhythms of plasma growth hormone, thyrotropin and thyroid hormones in prepuberty. Clinical endocrinology.

[11] Andersen S, Bruun NH, Pedersen KM, Laurberg P (2003). Biologic variation is important for interpretation of thyroid function tests. Thyroid: official journal of the American Thyroid Association.

[12] Hassi J, Sikkila K, Ruokonen A, Leppaluoto J (2001). The pituitary-thyroid axis in healthy men living under subarctic climatological conditions. The Journal of endocrinology.

[13] Reed HL (1995). Circannual changes in thyroid hormone physiology: the role of cold environmental temperatures. Arctic medical research.

[14] Levy SB (2013). Seasonal and socioeconomic influences on thyroid function among the Yakut (Sakha) of Eastern Siberia. American journal of human biology: the official journal of the Human Biology Council.

[15] Coomans CP (2015). Plasticity of circadian clocks and consequences for metabolism. Diabetes, obesity & metabolism.

[16] Coomans CP, Ramkisoensing A, Meijer JH (2015). The suprachiasmatic nuclei as a seasonal clock. Frontiers in neuroendocrinology.

[17] Follett BK (2015). “Seasonal changes in the neuroendocrine system”: some reflections. Frontiers in neuroendocrinology.

[18] Helm B (2013). Annual rhythms that underlie phenology: biological time-keeping meets environmental change. Proceedings. Biological sciences.

[19] Hut RA, Dardente H, Riede SJ (2014). Seasonal timing: how does a hibernator know when to stop hibernating?. Current biology: CB.

[20] Tamai TK, Yoshimura T (2017). Molecular and Neuroendocrine Mechanisms of Avian Seasonal Reproduction. Advances in experimental medicine and biology.

[21] Nicolau GY (1992). Chronobiology of pituitary-thyroid functions. Romanian journal of endocrinology / sponsore [sic] by the Academy of Medical Sciences.

[22] Barchetta I (2015). TSH levels are associated with vitamin D status and seasonality in an adult population of euthyroid adults. Clinical and experimental medicine.

[23] Ford HC, Johnson LA, Feek CM, Newton JD (1991). Iodine intake and the seasonal incidence of thyrotoxicosis in New Zealand. Clinical endocrinology.

[24] Moreno-Reyes R (2011). Seasons but not ethnicity influence urinary iodine concentrations in Belgian adults. European journal of nutrition.

[25] Nelson M, Quayle A, Phillips DI (1987). Iodine intake and excretion in two British towns: aspects of questionnaire validation. Human nutrition. Applied nutrition.

[26] Clerico Aldo, Trenti Tommaso, Aloe Rosalia, Dittadi Ruggero, Rizzardi Sara, Migliardi Marco, Musa Roberta, Dipalo Mariella, Prontera Concetta, Masotti Silvia, Musetti Veronica, Tozzoli Renato, Padoan Andrea, Bagnasco Marcello (2018). A multicenter study for the evaluation of the reference interval for TSH in Italy (ELAS TSH Italian Study). Clinical Chemistry and Laboratory Medicine (CCLM).

[27] Dittadi R (2017). Multicenter evaluation of the new immunoassay method for TSH measurement using the automated DxI platform. Clinica chimica acta; international journal of clinical chemistry.

[28] Rawlins ML, Roberts WL (2004). Performance characteristics of six third-generation assays for thyroid-stimulating hormone. Clinical chemistry.

[29] Jonklaas J (2014). Guidelines for the treatment of hypothyroidism: prepared by the american thyroid association task force on thyroid hormone replacement. Thyroid: official journal of the American Thyroid Association.

[30] Floriani C, Gencer B, Collet TH, Rodondi N (2018). Subclinical thyroid dysfunction and cardiovascular diseases: 2016 update. European heart journal.

[31] Halberg J, Halberg E, Regal P, Halberg F (1981). Changes with age characterize circadian rhythm in telemetered core temperature of stroke-prone rats. Journal of gerontology.

[32] Harrop JS, Ashwell K, Hopton MR (1985). Circannual and within-individual variation of thyroid function tests in normal subjects. Annals of clinical biochemistry.

[33] Sackett-Lundeen L (1990). Circadian and seasonal variation in iodine excretion in children in an endemic goiter area. Progress in clinical and biological research.

[34] Smals AG, Ross HA, Kloppenborg PW (1977). Seasonal variation in serum T3 and T4 levels in man. The Journal of clinical endocrinology and metabolism.

[35] Srivastava R (2010). Reflex and reflective testing: efficiency and effectiveness of adding on laboratory tests. Annals of clinical biochemistry.

[36] Santillo A (2012). Thyroid hormone receptor-beta gene expression in the brain of the frog Pelophylax esculentus: seasonal, hormonal and temperature regulation. General and comparative endocrinology.

[37] Inouye ST, Kawamura H (1979). Persistence of circadian rhythmicity in a mammalian hypothalamic “island” containing the suprachiasmatic nucleus. Proceedings of the National Academy of Sciences of the United States of America.

[38] Abe K, Kroning J, Greer MA, Critchlow V (1979). Effects of destruction of the suprachiasmatic nuclei on the circadian rhythms in plasma corticosterone, body temperature, feeding and plasma thyrotropin. Neuroendocrinology.

[39] Ebling FJ (2015). Hypothalamic control of seasonal changes in food intake and body weight. Frontiers in neuroendocrinology.

[40] Ikegami K, Yoshimura T (2017). The hypothalamic-pituitary-thyroid axis and biological rhythms: The discovery of TSH’s unexpected role using animal models. Best practice & research. Clinical endocrinology & metabolism.

[41] Santi D (2018). Seasonal variation of semen parameters correlates with environmental temperature and air pollution: A big data analysis over 6 years. Environmental pollution (Barking, Essex: 1987).

[42] Santi D (2016). Sperm quality and environment: A retrospective, cohort study in a Northern province of Italy. Environmental research.

[43] Follett BK, Nicholls TJ (1985). Influences of thyroidectomy and thyroxine replacement on photoperiodically controlled reproduction in quail. The Journal of endocrinology.

[44] Dawson A, King VM, Bentley GE, Ball GF (2001). Photoperiodic control of seasonality in birds. Journal of biological rhythms.

[45] Caldarelli G, Troiano G, Rosadini D, Nante N (2017). Adoption of TSH Reflex algorithm in an Italian clinical laboratory. Annali di igiene: medicina preventiva e di comunita.

