# Supplementary material for: Semi-annual seasonal pattern of serum thyrotropin in adults
Source: Sci Rep. 2019 Jul 25;9:10786. doi: 10.1038/s41598-019-47349-4 (PMC6658473; doi:10.1038/s41598-019-47349-4)

Semi-annual seasonal pattern of serum thyrotropin in adults

Authors: Daniele Santi[1,2], Giorgia Spaggiari[1,2], Giulia Brigante[1,2], Monica Setti[3], Simonetta Tagliavini[4], Tommaso Trenti[4], Manuela Simoni[1,2]

Affiliations: [1]Unit of Endocrinology, Department of Biomedical, Metabolic and Neural Sciences, University of Modena and Reggio Emilia, Modena, Italy. [2]Unit of Endocrinology, Department of Medical Specialties, Azienda Ospedaliero-Universitaria of Modena, OCSAE, Modena, Italy. [3]Service of Clinical Engineering, Azienda Ospedaliero-Universitaria of Modena, Modena, Italy. [4]Department of Laboratory Medicine and Anatomy Pathology, Azienda USL of Modena, Italy.

Address for Correspondence:

Daniele Santi, MD, PhD

Unit of Endocrinology, OCSAE

Via P. Giardini 1355, 41126, Modena, Italy,

Telephone: +39 0593961271, mail: daniele.santi@unimore.it

Supplementary figures legend

Supplementary figure 1. Thyroid-stimulating hormone serum levels frequency, considering the entire dataset.

Supplementary figure 2. Thyroid-stimulating hormone distribution analysis using ARIMA model (0,0,0), considering only data included in the normal laboratory range (0.35-4.94 microIU/mL). The first row shows data distribution; the second row highlights the seasonal pattern of data distribution; the third row shows the data trend across years and the last row the residuals distribution.

Supplementary figure 1.

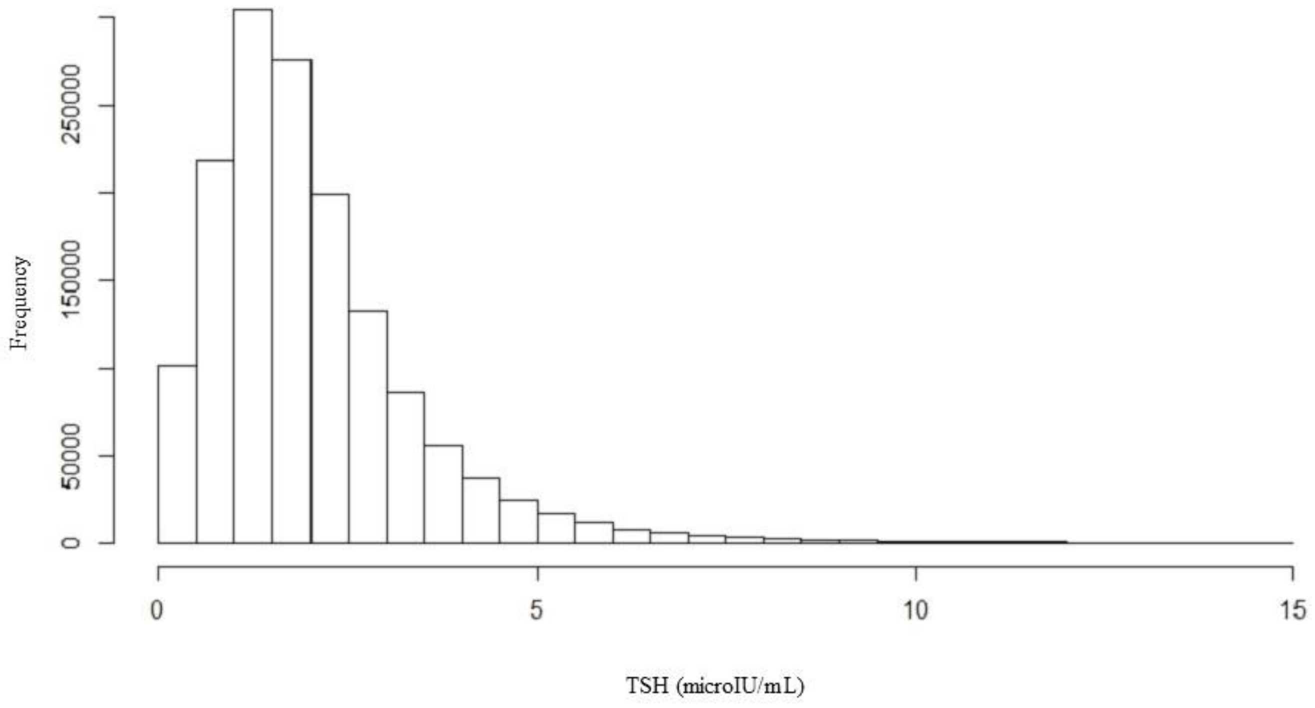

Supplementary figure 2.

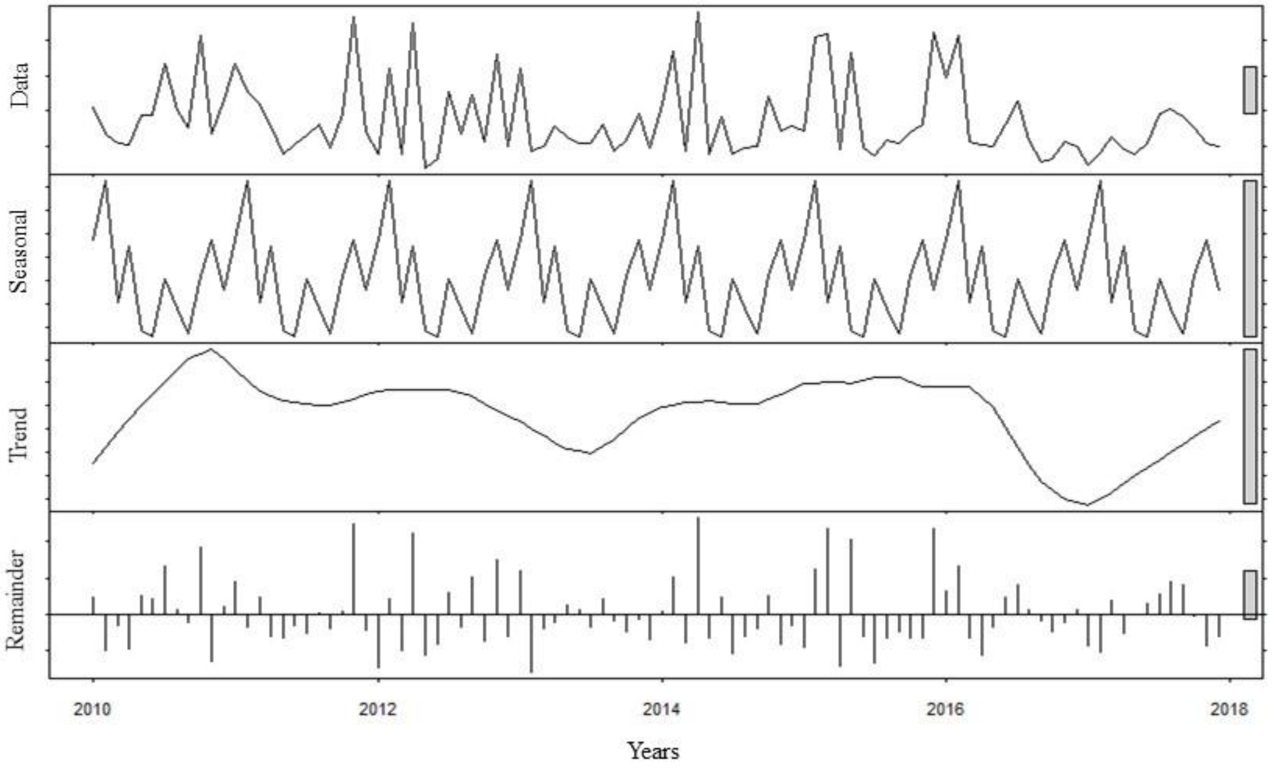

Supplement: Supplementary file 1 — Supplementary figures 1 and 2 [file 41598_2019_47349_MOESM1_ESM.pdf]
